# Novel innate cancer killing activity in humans

**DOI:** 10.1186/1475-2867-11-26

**Published:** 2011-08-03

**Authors:** Michael J Blanks, John R Stehle, Wei Du, Jonathan M Adams, Mark C Willingham, Glenn O Allen, Jennifer J Hu, James Lovato, Istvan Molnar, Zheng Cui

**Affiliations:** 1 Molecular Genetics & Genomics Program, Wake Forest University School of Medicine, Medical Center Boulevard, Winston-Salem, North Carolina, 27157, USA; 2 Department of Pathology -Tumor Biology, Wake Forest University School of Medicine, Medical Center Boulevard, Winston-Salem, North Carolina, 27157, USA; 3 Deparment of Biostatistical Sciences, Wake Forest University School of Medicine, Medical Center Boulevard, Winston-Salem, North Carolina, 27157, USA; 4 Department of Cancer Biology, Wake Forest University School of Medicine, Medical Center Boulevard, Winston-Salem, North Carolina, 27157, USA; 5 Department of Hematology & Oncology, Wake Forest University School of Medicine, Medical Center Boulevard, Winston-Salem, North Carolina, 27157, USA; 6 Department of Epidemiology and Public Health, University of Miami, Miller School of Medicine, Miami, Florida, 33136, USA

## Abstract

**Background:**

In this study, we pilot tested an in vitro assay of cancer killing activity (CKA) in circulating leukocytes of 22 cancer cases and 25 healthy controls.

**Methods:**

Using a human cervical cancer cell line, HeLa, as target cells, we compared the CKA in circulating leukocytes, as effector cells, of cancer cases and controls. The CKA was normalized as percentages of total target cells during selected periods of incubation time and at selected effector/target cell ratios in comparison to no-effector-cell controls.

**Results:**

Our results showed that CKA similar to that of our previous study of SR/CR mice was present in human circulating leukocytes but at profoundly different levels in individuals. Overall, males have a significantly higher CKA than females. The CKA levels in cancer cases were lower than that in healthy controls (mean ± SD: 36.97 ± 21.39 vs. 46.28 ± 27.22). Below-median CKA was significantly associated with case status (odds ratio = 4.36; 95% Confidence Interval = 1.06, 17.88) after adjustment of gender and race.

**Conclusions:**

In freshly isolated human leukocytes, we were able to detect an apparent CKA in a similar manner to that of cancer-resistant SR/CR mice. The finding of CKA at lower levels in cancer patients suggests the possibility that it may be of a consequence of genetic, physiological, or pathological conditions, pending future studies with larger sample size.

## Background

It has been hypothesized for more than 100 years that cancer cells develop frequently in healthy individuals due to the constant cell-damaging factors they are exposed to from the environment [1].

Therefore, in order to remain healthy (cancer free), there must be a naturally existing system for continuously identifying and removing cancer cells before they accumulate into detectable cancer lesions [2-4]. This system, termed cancer immunosurveillance, is believed to be critical in protecting hosts and its failure can lead to malignancy. Further support of this system came through the serendipitous discovery of the Spontaneous Regression/Cancer Resistant (SR/CR) mouse [5].

Multiple human studies have investigated the relationship between cancer and the immune system [6-10]. For example in 2000, Imai et al. reported the results of an eleven-year prospective cohort study among 3,625 Japanese individuals [7]. Specifically, this study compared the cytotoxic function of Natural Killer (NK) cells by means of a ^51^Chromium release assay and an extensive follow-up investigation of cancer incidence among the same individuals. Their findings suggested that individuals who possessed high to medium levels of NK-mediated natural cytotoxicity could have a reduced risk of cancer incidence later in life. In 1984, Strayer et al. reported studies of the possible role of cell-mediated toxicity and familial cancer incidence [10]. Like the methods of Imai, Strayer's also included the use of a ^51^Chromium release assay to measure immunological function. Results among 79 normal individuals in Strayer's studies showed that natural cell-mediated cytotoxicity inversely correlated with the incidence of family members with cancer, thus, implicating their assay as a potential marker of familial cancer risk. These findings underscore the powerful diagnostic and prognostic tools that can potentially arise from studies intended to observe the immune system's ability to fight cancer. However, the relevance of these assays is difficult to verify without the ability to either directly challenge the sampled individual or observe this individual throughout their lifetime for the development of cancer. A human adaptation of the CKA assay may be preferential to previous methods, because it has been developed and validated under conditions defined by parallel observations within an animal model of transferrable, heritable, innate cancer resistance [11,12].

Our laboratory previously described the unique, heritable phenotype of cancer resistant (SR/CR) mice [5]. The SR/CR mice are capable of resisting large doses of highly lethal cancers [5,11,13]. Our observations further demonstrated that SR/CR cancer resistance is mediated by a massive infiltration of predominantly innate immune cells, which migrate directly to the site of a tumor challenge. When observed *in vitro*, SR/CR immune cells also demonstrate an innate, anti-cancer cytotoxic function that is significantly superior to that of the immune cells of wild type mice [5]. Remarkably, this anti-cancer cellular activity is capable of being transferred to non-resistant, wild-type mice, where it has been shown to provide therapeutic resistance in a model of established cancer [11]. The ability of SR/CR leukocytes to mediate an enhanced innate resistance to cancer is a trait that enabled the development of the previously described screening assay [11]. Based on this innate phenotype, we previously developed an *in vitro *cancer cell killing assay that recapitulated the *in vivo *activity of leukocytes. In this assay, cancer cells were co-incubated with immune (effector) cells to determine their anti-cancer activity. Through the development and use of this assay, naïve leukocytes that demonstrated high levels of *in vitro *cancer killing activity (CKA) displayed predictive values that were capable of distinguishing which mice among litters would survive a direct challenge of cancer *in vivo *[11]. Given the predictive value of this assay relative to SR/CR mice and corresponding therapeutic potential, along with the inherently low risk involved with leukocyte collection for *in vitro *screening, we set out to determine if similar CKA could be observed from isolated human leukocytes. Given that our assay was evaluated with the guidance of the SR/CR mouse phenotype, we suspect that if any CKA is to be observed in humans, its value may eventually provide more relevant insights when compared to conventional methods. Additionally, we collected data of the leukocytes of participants with cancer. These findings provided a foundation for future studies regarding cancer subtype and assay conditions. The implications of these findings are intended to provide an early milestone, as we search for ways the SR/CR-based findings can have therapeutic relevance for humans.

## Methods

### Study Design

A modified cytotoxicity assay and the existence of CKA in the leukocytes of humans were investigated in a randomized case-control pilot study. In addition, the variation of CKA within three healthy individuals over time was investigated in an initial set of optimization studies.

### Participant Recruitment

Between April of 2006 and July of 2007, 48 study participants were recruited among the Wake Forest University Baptist Medical Center Comprehensive Cancer Center as cancer patients or otherwise healthy individuals. All participants were entered into the study under approval and guidance obtained from the Wake Forest University Institutional Review Board. Participant selection criteria for controls included individuals with: 1) no serious medical or psychiatric condition that would prohibit consent and sample collection, 2) no personal history of cancer, and 3) no history of immunosuppressive therapy or radiation therapy for any disease. Participant selection criteria for cases included individuals with: 1) no serious medical or psychiatric condition that would prohibit consent or sample collection, 2) a diagnosis of metastatic cancer, and 3) no history of chemotherapy or radiation therapy within three months prior to the sampling date. In addition to health status, demographic information of participants including age, gender, and ethnicity was collected.

### Sample Procurement and Processing

From each participant, 24-30 mL of blood was collected by venipuncture into three Vacutainer^® ^CPT^® ^Cell Preparation Tubes with Sodium Citrate (Becton, Dickinson and Company, Franklin Lakes, NJ). Samples were labeled with a coded study number by a clinical coordinator, and transported immediately to an on-site processing facility. Upon receipt, coded samples were processed according to the manufacture's instructions, thereby producing a fraction of mononuclear (MNC) leukocytes from each sample. Viable leukocytes were quantified with a haemocytometer based on ability to exclude trypan blue dye.

### Cancer Target Maintenance

The HeLa cell line, which originated from human cervical adenocarcinoma, was chosen as the target for use in the assay (American Type Tissue Culture Collection, Manassas, VA). HeLa cells were maintained using standard tissue culture methods. Briefly, HeLa cells were grown and maintained at 37°C, 8% CO_2_, in T75 cm^2 ^cell culture flasks in Dulbecco's Modified Eagle's Medium (D-MEM) (Invitrogen, Carlsbad, CA) supplemented with the following ingredients: 10% volume/volume Fetal Bovine Serum (FBS) (Sigma. St. Louis, MO), Penicillin (Sigma. St. Louis, MO), Streptomycin (Sigma. St. Louis, MO) & supplemental L-glutamine (Sigma. St. Louis, MO). HeLa cell culture were split and passaged before reaching 70% surface confluence in culture flasks.

### Leukocyte-mediated Cancer Killing Activity (CKA) Assay

CKA was determined through the use of a modified form of the SR/CR *in vitro *cytotoxicity assay, published previously [5,11]. Incubation times were either 12 hr or 24 hr, while most presented data came from 24 hr incubation. The effector to target ratio used in these studies ranged between 19 to 28 effectors per target. These conditions were emulated from the mouse-specific CKA assay, while items including tumor target selection and effector sample collection were altered in order to adapt the assay for use in humans. Briefly, isolated leukocytes were co-incubated in BD Falcon 35 mm^2 ^cell culture dishes (Becton-Dickinson, Franklin Lakes, NJ) with adherent HeLa tumor targets at desired effector to target ratios. HeLa targets were allowed to reach approximately 60-80% confluence at 37°C prior to effector addition. Following effector addition, all assays including controls were carried out at 39°C. Each data point represented duplicates or triplicates of the same assay. In parallel wells, duplicate HeLa cells were incubated alone without effector cells as controls. Following incubation, samples were washed twice with Phosphate Buffered Saline (PBS) to remove leukocytes and dead or dying target cells that were detached from culture surfaces. Remaining live adherent target cells were treated with PBS containing 0.25% trypsin and 0.05% EDTA. Trypsinization was halted with the addition of culture media containing 10% fetal bovine serum. Samples were centrifuged at 400 × g for 5 min and resuspended in ice-cold PBS. Trypan-blue-excluding cells were quantified with a haemocytometer.

### Statistical Analyses

CKA was defined as the arithmetic difference in the number of trypan-blue-negative cells in the control group from the number of trypan-blue-negative cells in the experimental group, expressed as a percentage of the number of trypan blue negative cells in the control group:

Student's *t*-test (for Total), χ^2 ^tests, and two way ANOVA were used to compare control and case demographic variables. Binary logistic regression was used to calculate odds ratios (OR) and 95% confidence intervals (CI) for the association between CKA activity and cancer risk. Statistical analyses were completed using SPSS v. 17.0 (SPSS, Chicago, IL).

## Results

### Cancer Killing Activity and Participant Demographics

Table 1 summarizes the demographic characteristics of cases and controls. The mean and standard deviation of CKA are also presented. There were no significant differences in the distribution of controls and cases by gender, ethnicity, or age. Overall, CKA was higher in controls (mean = 46.28; SD = 27.22) compared to cases (mean = 36.97; SD = 21.39), although the results did not reach statistical significance (*p *= 0.20). Males had significantly higher CKA compared to females (ANOVA, *p *= 0.001). This relationship was apparent among both cases and controls, and subsequently investigated in an association study of cancer risk (see Table 2). There was no significant difference in CKA by ethnicity or age.

**Table 1:** Cancer Killing Activity (CKA) in Controls and Cases by Gender, Race, and Age

Variable	Controls		Cases		Chi-Square p	ANOVA p
	N (%)	Mean (SD)	N (%)	Mean (SD)		
Total	25 (53)	46.28 (27.22)	22 (47)	36.97 (21.39)		0.20
Gender						
Female	17 (68)	37.20 (23.85)	13 (59)	28.83 (16.15)		
Male	8 (32)	65.57 (24.76)	9 (41)	48.72 (23.39)	0.61	0.001
Race						
White	24 (96)	47.73 (26.79)	20 (95)	36.11 (21.53)		
Black	1 (4)	11.42	1 (5)	54.95	0.93	0.62
Age						
< 60	14 (56)	38.43 (24.30)	8 (36)	34.06 (15.18)		
≥ 60	11 (44)	56.28 (28.53)	14 (64)	38.63 (24.63)	0.18	0.13

**Table 2:** Association Between CKA Activity and Cancer Risk

Gender	Controls N (%)	Cases N (%)	OR (95% CI)	Adj. OR (95% CI)^2^
(A) Females				
≥ 50.0^1^	7 (41)	2 (15)		
< 50.0	10 (59)	11 (85)	3.85 (0.64, 23.05)	
(B) Males				
≥ 50.0	6 (75)	4 (44)		
< 50.0	2 (25)	5 (56)	3.75 (0.47, 29.75)	
(C) Total				
≥ 50.0	13 (52)	6 (29)		
< 50.0	12 (48)	15 (71)	2.89 (0.85, 9.82)	**4.36 (1.06, 17.88)**

^1 ^The cutoff value based on the median CKA activities of all controls

^2 ^OR adjusted for gender and age (≥ 50, < 50)

### Association of Cancer Killing Activity and Cancer Risk

The association between CKA and cancer risk is shown in Table 2. The cutoff value (50.0) is based on the median value of the control group to divide subjects into dichotomous groups. (A) Females with low CKA had a higher cancer risk (OR = 3.85, 95% CI = 0.64, 23.05), although these results did not approach significance. (B) Males with low CKA had nearly a four fold but not significant increase in cancer risk (OR = 3.75, 95% CI = 0.47, 29.75). The small subgroup sample size could be a contributing factor for these results not approaching significance. (C) Total population with low CKA had a significant increased cancer risk (OR = 4.36, 95% CI = 1.06, 17.88) after adjusting for gender and age.

### Sample Variation of CKA among Healthy Individuals Over Time

Figure 2 provides a summary of a preliminary investigation regarding the variation of CKA among three healthy individuals, over time.

## Discussion and Conclusions

**Figure 1:**
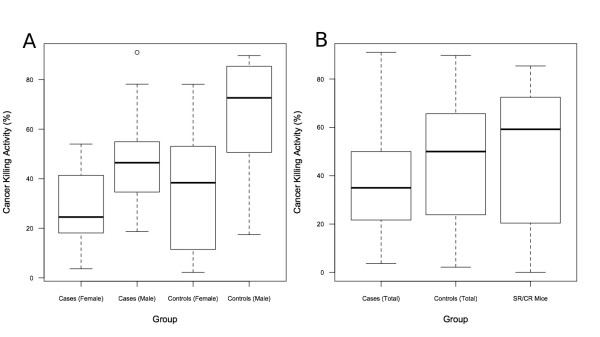
**Distribution of CKA among subgroups**. Figure 1 shows a box plot representing the CKA of seven groups. The top and bottom portions of the boxes represent the 75^th ^and 25^th ^percentiles, whereas the lines in the middle of the boxes represent the median or 50^th ^percentile of the CKA distribution. The whiskers represent the upper and lower adjacent values. **A) **Subject groups are defined as: female cases (median = 24.5), male cases (median = 46.5), female controls (median = 38.4), and male controls (median = 72.7). **B) **Summary distribution of CKA values from all cases (median = 35.0) and controls (median = 50.0) as well as CKA measures obtained from thioglycollate-elicited peritoneal infiltrating leukocytes of SR/CR mice against S180 tumor targets (median 59.2).

Overall, these results suggest that human leukocytes possess varying levels of CKA. Additionally, several intriguing findings were obtained from our initial comparison of CKA among healthy participants or those with cancer. The results of an association study between cancer risk and CKA presented significant findings when adjusting for an observed gender bias (Table 2). Specifically, these findings suggested that participants with cancer were 4.36 times more likely to possess effector leukocytes that displayed a CKA below 50% against HeLa cancer targets, *in vitro*. Also, although not significantly different, the sample mean CKA value among cases was lower than that of controls. The result of this comparison was also displayed within demographic subgroups of gender and age (Table 1). Given the range of CKA values collected, these levels appear to be comparable to historical CKA levels of greater than 50% among SR/CR mouse leukocytes (Figures 1A and 1B) [5]. Moreover, these results suggest that components of cancer resistance, such as enhanced immune function witnessed in SR/CR mice, may also be detectable through the use of a similar human *in vitro *assay described here.

**Figure 2:**
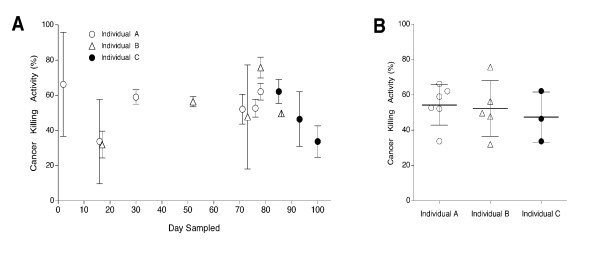
**Sample Variation and Presence of CKA over time**. Figure 2 represents the results of repeated measures of CKA over time in each of three healthy individuals. **A) **Mean CKA values, along with standard deviations were collected, analyzed and summarized by day of collection, per individual. **B) **A summary of these values is presented as the distribution of CKA among each individual. Horizontal bars indicate mean CKA values, along with standard deviation per individual.

Upon review of these findings, several considerations may aid in the future development of the *in vitro *cytotoxicity assay for humans. One such condition is apparent upon inspection of the distribution of CKA among cases versus controls. As illustrated in Figure 1B, the majority of CKA values among cases are less than 50%, whereas CKA values measured from controls display larger variation. Generally, controls presented CKA values that were either high or low, whereas cases more commonly presented low CKA values. This relationship is affirmed by a consistent finding among SR/CR mice in regards to the *in vitro *cytotoxicity assay. As previously reported in 2006, Hicks et al. demonstrated that the use of the *in vitro *cytotoxicity assay could serve as a predictive indicator for the SR/CR phenotype among naïve pups [11]. These results showed that 84% of mice with CKA values greater than 50% were later found to be cancer resistant (SR/CR). Furthermore, 100% of mice with CKA values less than 50% were later found to die from the same cancer challenge. These findings suggest that measured values of *in vitro *CKA are consistently low in susceptible mice. When these historical findings in SR/CR mice are combined with observations from the human results in this study, an intriguing pattern arises regarding the accuracy of the *in vitro *cytotoxic assay. Since an otherwise healthy individual could possess low immunological function, the possibility arises that CKA may be a dynamic value among individuals and possibly more sensitive than we originally anticipated. The evaluation of conditions that affect the stability and sensitivity of this assay is an ongoing subject of our investigation, specifically by consistently measuring CKA in the same individual over an extended time frame. Figures 2A and 2B demonstrate early results of our ongoing preliminary investigation, in which we repeatedly sampled the same healthy individuals. Results of these studies indicate that CKA is persistent among individuals, and warrants future investigation in order to generate a CKA baseline for each individual. Eventually such a measurement may be capable of reflecting the transient nature of the immunological anti-cancer capacity within a healthy individual. The changes in CKA could coincide with transient influencing factors, such as stress, or otherwise unforeseen immunological suppression due to infection or disease [14-18]. Pending future studies, the use of CKA may serve as a functional marker for immunological status thereby serving as a risk factor for diseases that are dependant on immunological function, such as infection and cancer.

Given the relatively small study size, we suspect that the heterogeneity of CKA among specific cancer subtypes may also contribute to the statistically insignificant difference between total sample means (Table 1 and Figure 1B). Eligibility criteria within the context of this study required patients to have metastatic disease and did not limit inclusion to one specific type of cancer diagnosis. An *ad hoc *analysis of patient diagnosis did not reveal a clear trend among individuals, as most cancer origin subtypes were only represented once among cases. In response to this dilemma, we intend to expand the cancer targets being assessed for CKA per patient. Ideally, cancer targets that are more appropriately representative of the disease subtype of an individual would more accurately reflect the immune system's contribution to the individual's disease as determined by the *in vitro *assay. Additionally, results of such panels may eventually help to identify potential leukocyte effector subsets that have a more profound effect on specific cancer types. It is of great interest to investigate CKA among multiple leukocyte subtypes in order to determine their independent effects as well as to identify the optimal effector population subsets that could be targeted for therapeutic use in the future. Current efforts are underway by our laboratory to develop the *in vitro *cytotoxicity assay for use in a high-throughput technology. Such technology would increase experimental efficiencies, which would help in addressing conditional variations in ongoing experiments with the goal of answering these many developing questions.

Patient demographics were also sampled in this study, illustrating that gender alone demonstrated a significant difference in CKA among both cases and controls (Table 1). This observation led to an association-adjustment due to gender and age bias between cancer risk and CKA (Table 2). A clear reasoning as to why gender would display this affect on CKA remains unknown, yet gender-specific trends regarding cancer incidence are reported and well established [19,20]. This result is in agreement with the cytotoxic measures witnessed by Imai et al., showing that among all individuals across all age groups, cytotoxicity was detected at higher levels in leukocytes from men rather than in women [7]. An obvious difference in women that could possibly explain the modulation of immune responses is the presence of elevated estrogen levels relative to men [21]. Estrogen has been shown to suppress NK-mediated cytotoxicity in a dose dependent manner [22]. Also, estrogen effects on macrophages have been shown to reduce TNFα expression, due to the inhibition of transcription factors by the estrogen-ER complex [23]. Future correlative investigations regarding the level of estrogen or other gender-specific hormones may be of value to discern why women have lower levels of CKA.

Age, when divided into two groups: a) greater than, or b) less than 60 years of age, showed no significant differences among both cases and controls (Table 1). This finding suggests that age did not show differences in CKA observed among cases and controls. This was an anticipated finding given that cases were matched with similarly aged controls when possible by the clinical coordinator. Future studies that specifically target age as a discerning factor would be of value given the relationship in humans between cancer incidence, mortality and age [5,11-13,24-27]. Results regarding ethnicity remain inconclusive in this study, as sufficient representative sampling did not occur. In the future, it will be a crucial requirement to our understanding of CKA to study ethnicity-based values in humans, as incidences of some forms of cancer are known to occur in disparity between specific populations [20,28,29].

In order to translate the anti-cancer therapeutic effect, which has previously been demonstrated by SR/CR leukocyte adoptive transfers in mice [11], it is imperative that we identify biological markers of this unique phenotype in humans. The existence of CKA in humans, as we have demonstrated here, is an initial step towards that goal.

## List of Abbreviations

CKA: Cancer Killing Activity; NK: Natural Killer; MNC: Mononuclear Leukocyte; SR/CR: Spontaneous Regression/Cancer Resistant

## Competing interests

The authors declare that they have no competing interests.

## Authors' contributions

MJB was responsible for data collecting, processing and manuscript writing. JRS was responsible for performing a portion of the studies, discussing the study design and manuscript writing. WD was responsible for performing the majority of assays and developing the assay method. JMA was responsible for performing a portion of the studies and discussing the study design. MCW was responsible for study design, data collection, study discussion and manuscript writing. GOA was responsible for data analysis. JJH was responsible for data analysis, study discussion and manuscript writing. JL was responsible for a portion of the study design. IM was responsible for study design, conceptualization of study ideas, IRB application and patient recruitment.

ZC was responsible for the entire project including conceptualization of study ideas, study design, evaluating results, recruiting the study team, study discussion, data analysis, manuscript writing and funding the study.

The authors read and approved the manuscript
